# Effect of tea saponin on ephyrae and polyps of the moon jellyfish *Aurelia* sp.1

**DOI:** 10.1371/journal.pone.0182787

**Published:** 2017-08-04

**Authors:** Zhijun Dong, Tingting Sun, Likun Liang, Lei Wang

**Affiliations:** 1 Key Laboratory of Coastal Zone Environmental Processes and Ecological Remediation, Yantai Institute of Coastal Zone Research, Chinese Academy of Sciences, Yantai, Shandong, P.R., China; 2 College of Life Sciences, Yantai University, Yantai, Shandong, P.R., China; Evergreen State College, UNITED STATES

## Abstract

The moon jellyfish (*Aurelia* sp.1) is thought to be a nuisance for the sea cucumber aquaculture, which commonly occur in the sea cucumber (*Apostichopus japonicus*) culture ponds of the Yellow Sea, China. To develop an appropriate method to control *Aurelia* sp.1 blooms, the toxic effects of tea saponin on *Aurelia* sp.1 ephyrae and polyps were tested in laboratory experiments. Our results revealed that tea saponin caused significant morphological changes, behavioral abnormality and mortality in *Aurelia* sp.1 ephyrae and polyps in 24 h and 48 h exposure experiments. The 24 h and 48 h median lethal concentrations (LC_50_) values of tea saponin for *Aurelia* sp.1 ephyrae were 1.9 and 1.1 mg L^-1^ respectively, while the LC_50_ value for *Aurelia* sp.1 polyps was 0.4 mg L^-1^ after 24h and 48 h of exposure to tea saponin. Comparison with literature results of tea saponin on *A*. *japonicus* indicates that the resistance of *A*. *japonicus* to tea saponin is 12–18 times greater than that of *Aurelia* sp.1 ephyrae. Therefore, the appropriate tea saponin dosage for the control of *Aurelia* sp.1 should be paid enough attention in order to minimize possible damage for sea cucumber. We suggest that the recommended level of tea saponin to eradicate *Aurelia* sp.1 ephyrae and polyps in sea cucumber culture ponds be lower than 1.35 mg L^-1^.

## Introduction

The moon jellyfish *Aurelia* spp. (Cnidaria: Scyphozoa) are the most common scyphozoan species, and have a wide geographic distribution in coastal waters [1]. Global phylogenetic studies reveal at least 13 cryptic species in the genus *Aurelia*, and *Aurelia* sp.1 occurs in the major warm-temperate regions including China, Japan, Korea, Australia and California [2–4]. The *Aurelia* sp.1 blooms found in the coastal waters of China, Japan, and Korea have negatively affected coastal power plant operations, local fisheries and tourism [3, 5–6].

Recently, high densities of *Aurelia* sp.1 have occurred in sea cucumber (*Apostichopus japonicus*) culture ponds located on the coasts of the Yellow Sea, China, and are thought to be a nuisance to the sea cucumber [7]. In previous studies, *A*. *aurita* blooms have been observed to cause severe damage to the aquaculture industry [8–11]. For example, the ephyrae and small medusae of *A*. *aurita* are thought to have caused huge losses of farmed fish in Norway and Scotland through suffocation [8], and *A*. *aurita* can cause severe gill problems in farmed Atlantic salmon, *Salmo salar* [9–10]. Furthermore, the Chinese farmers have also observed abnormal response of *A*. *japonicus* to high density of *Aurelia* sp.1 ephyrae in the culture ponds. Therefore, it is necessary to develop an appropriate method to control the moon jellyfish *Aurelia* sp.1 in coastal sea cucumber culture ponds.

Tea seed cake is the residue left over after pressing oil from the seeds of *Camellia sinensis* and is composed of 10%–15% saponin [12], which is a tea seed-derived natural surfactant. It is composed of glycosides with aglycones related to either sterols or triterpenes, and the hydrophilic groups contain both hydroxy groups and ester groups [13]. Tea saponin has been reported to damage red blood cells and affect oxygen uptake and the level of hemoglobin in fish [14–15]. In aquaculture, tea seed cakes have commonly been used to remove the predators from culture farms [14, 16–19]. For example, Tang (1961) showed that the resistance of shrimp to saponin is approximately 50 times greater than that of fish and the results of Terazaki et al. (1980) indicated that crude saponin extracted from Thai tea seed was toxic to indigenous predatory fishes but less toxic to shrimp [16–17]. The results obtained by Zhu et al. (1991) indicated that tea saponin was strongly toxic to six species of harmful fish found in prawn ponds [18]. In addition, tea seed cake is also effective at removing the hydrozoan jellyfish (*Proboscidatyla ornata*) from shrimp and crab farms without harming the cultivated species [19].

Chinese farmers often use tea seed cakes to eradicate *Aurelia* sp.1 in sea cucumber culture ponds. However, the appropriate dosage of tea seed cake (tea saponin) for eradicating *Aurelia* sp.1 has not previously been known. In the current study, we attempted to examine the toxic effects of tea saponin on ephyrae and polyps of the harmful jellyfish, *Aurelia* sp.1. The purpose of our study was to provide quantitative information on the appropriate tea saponin dosage to eradicate the ephyrae and polyps of *Aurelia* sp.1 in sea cucumber culture ponds.

## Materials and methods

### Ethics statement

There were no specific permissions for catching the moon jellyfish *Aurelia* sp.1 in coastal waters of Rongcheng because it is thought to be a nuisance in Chinese Seas. The jellyfish samples do not involve endangered or protected species.

### Experimental organisms

Colonies of *Aurelia* sp.1 polyps attached to tubeworms were collected from a coastal lake in Rongcheng and transported to a laboratory at the Yantai Institute of Coastal Zone Research, Chinese Academy of Sciences. Polyps were placed in plastic tanks filled with filtered seawater in a low-temperature incubator that provided a constant temperature of 6°C (Yiheng, Shanghai), and they were fed newly hatched *Artemia salina* nauplii, twice weekly. After the polyps were fed for four hours, the seawater was replaced with filtered seawater. The polyps were maintained in the laboratory for about 4 months prior to the toxic experiments. Strobilation was induced by raising the temperature to 13°C. The released 1-day-old ephyrae were then poured into a beaker and immediately transferred into the wells of 24-well plates (Canvic, Shanghai) filled with 10 ml of tea saponin test solutions for the toxic experiments. Polyps of similar sizes were separated from the main cultures and placed in the wells of 24-well plates (Canvic, Shanghai) for re-attachment. After attachment, the plates containing polyps were filled with 10 ml of tea saponin test solutions in preparation for the toxic experiments.

### Experimental design and procedures

Triterpenoid saponin (60% w/w) extracted from tea (*Camellia sinensis*) seeds was purchased from Shanghai Yuanye Bio-Technology Co., Ltd and stored at 4°C until use. Tea saponin stock solutions were prepared by diluting 0.017 g of tea saponin with 100 ml of distilled water to obtain a concentration of 100 mg l^-1^. Tea saponin test solutions consisting of saponin concentrations of 0 (control), 0.1, 0.5, 1, 2, 3, 4, 5, 10 and 50 mg l^-1^ were prepared by diluting the appropriate amount of stock solution with 0.22 μm filtered seawater (salinity 31 ppt). For the toxicity experiments, three replicates were prepared for each concentration, and each replicate contained eight ephyrae or polyps that were each placed in individual wells to avoid interactions among the organisms [20–21].

Temperature and salinity were measured in a sea cucumber culture pond during an *Aurelia* sp.1 ephyrae bloom in 2016. The seawater temperature was 15.6°C and the salinity was 32.5 ppt. Therefore, similar environmental factors were used in our experiments. All of the plates were maintained at 16°C in an incubator in the dark (Boxun, Shanghai). The salinity and pH of the tea saponin test solutions were measured using a YSI-600 multi-parameter water quality monitor. After 24h and 48h of exposures, the acute and sub-lethal end-points were calculated. All of the ephyrae and polyps were also photographed using an Olympus SZX10 stereo microscope fitted with an Optec TP510 digital camera.

The condition of ephyrae was described using two indicators [20–21]: 1) ephyrae pulsation frequency, which was calculated by counting the number of pulsations (frequency of pulsation, Fp) made by each ephyra in 1 min; 2) immobility, which described ephyrae that were on the bottom of the wells and did not change their position for 5 s [22]. Both the immobility and frequency of pulsation measurements were evaluated by three-person panel to reduce errors.

The polyp condition was described using two indicators [23]: 1) unhealthiness, which was indicated by tentacle shrinkage or loss, deformities such as an unusually wide mouth or an extruded gastric cavity, paleness or inactivity; 2) mortality, which was indicated by a complete loss of tentacles, a mouth that was closed or unable to close, insecurity on the settling plate, or decomposition. The number of polyps showing signs of each indicator was determined.

### Data analysis

All of the experiments were conducted in triplicate, and the mean is presented. The 24h and 48h median lethal concentrations (LC_50_) or median effective concentrations (EC_50_) and the related 95% confidence limits (CL) of tea saponin were calculated by the probit method using the SPSS 19.0 PROBIT procedure [24]. One-way analysis of variance (ANOVA), followed by an LSD pair-wise comparison of the average pulsation frequency and immobility values for each of the treatment concentrations to the control values, was performed to calculate the lowest observed effect concentration (LOEC, P < 0.01) [21]. Homogeneity of variances was tested using the Levene test. All analyses were performed using the SPSS Statistics version 19 (IBM, Armonk, NY, USA).

## Results

Water quality parameters among the different treatment concentrations were determined at the beginning of our experiments. Salinity ranged from 31.50 ppt to 31.59 ppt, while pH ranged from 7.86 to 7.94. Seawater temperature was stable at 16°C (± 1°C) during the exposure to tea saponin.

**Fig 1:**
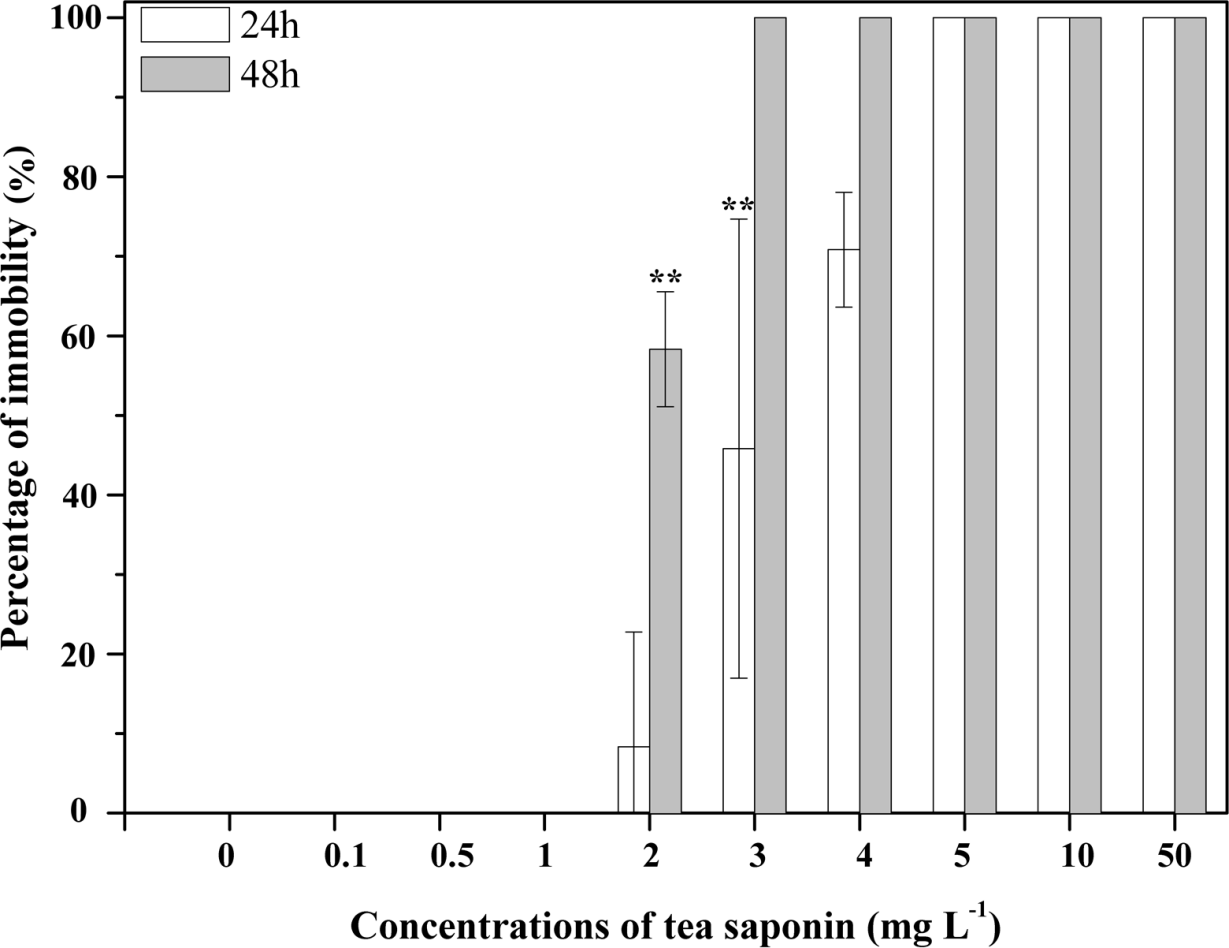
Immobility of 1-day-old *Aurelia* sp.1 ephyrae after exposure to increasing concentrations of tea saponin. Mean ± SE, n = 3; **P < 0.001.

**Fig 2:**
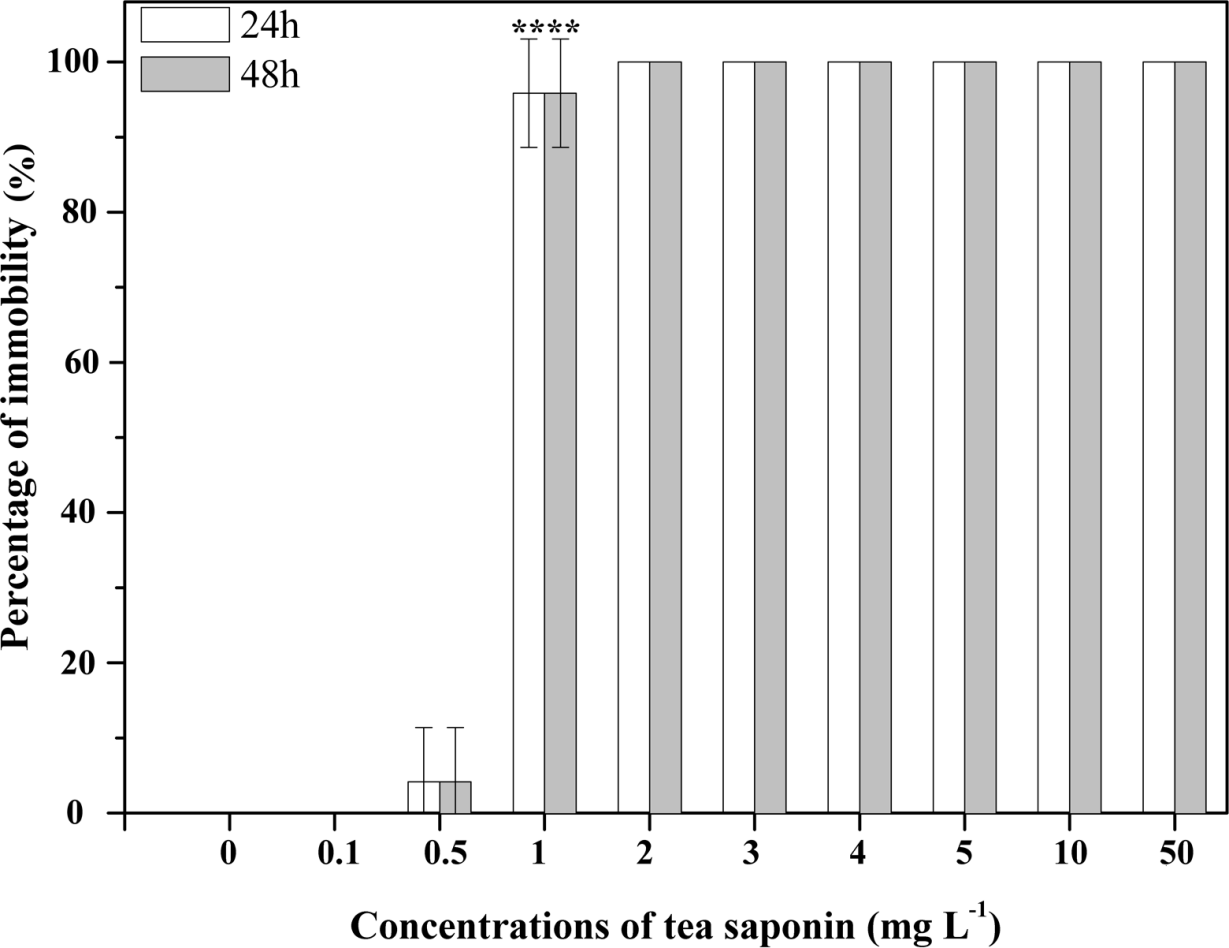
Mortality of *Aurelia* sp.1 polyps after exposure to increasing concentrations of tea saponin. Mean ± SE, n = 3; **P < 0.001.

**Fig 3:**
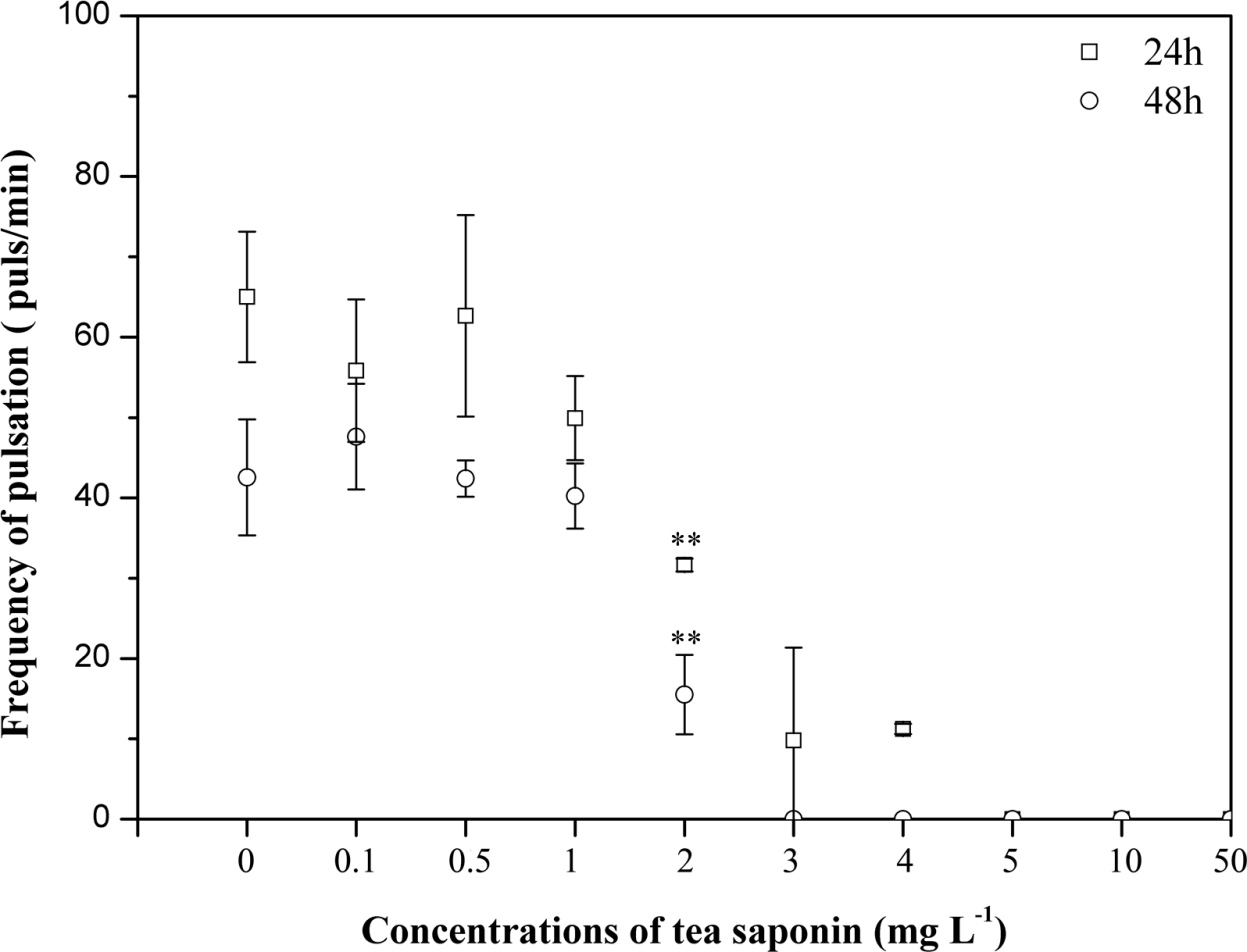
Pulsation frequencies of 1-day-old *Aurelia* sp.1 ephyrae after exposure to increasing concentrations of tea saponin. Mean ± SE, n = 3; **P < 0.001.

**Fig 4:**
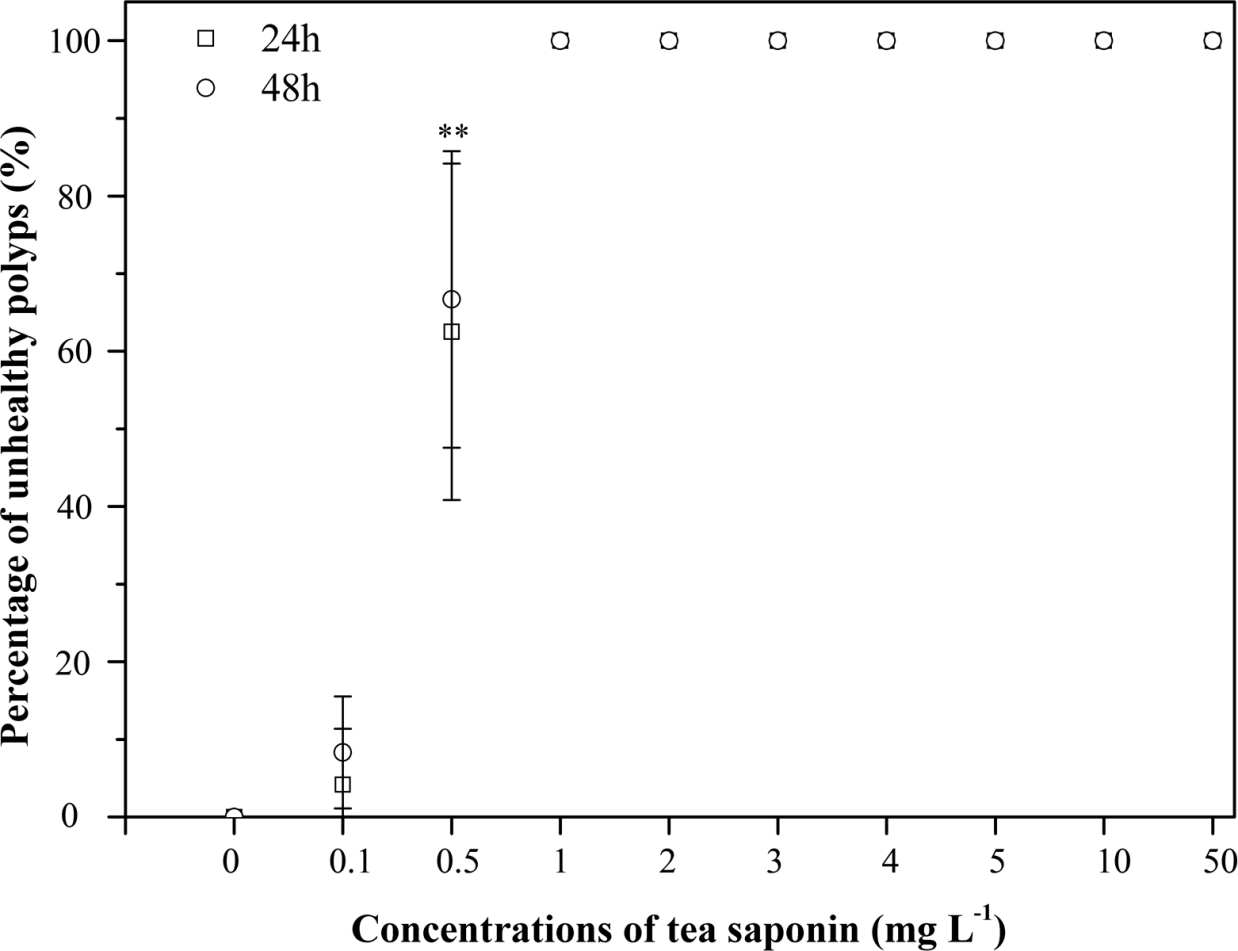
Percentage of unhealthy *Aurelia* sp.1 polyps after exposure to increasing concentrations of tea saponin. Mean ± SE, n = 3; **P < 0.001.

**Fig 5:**
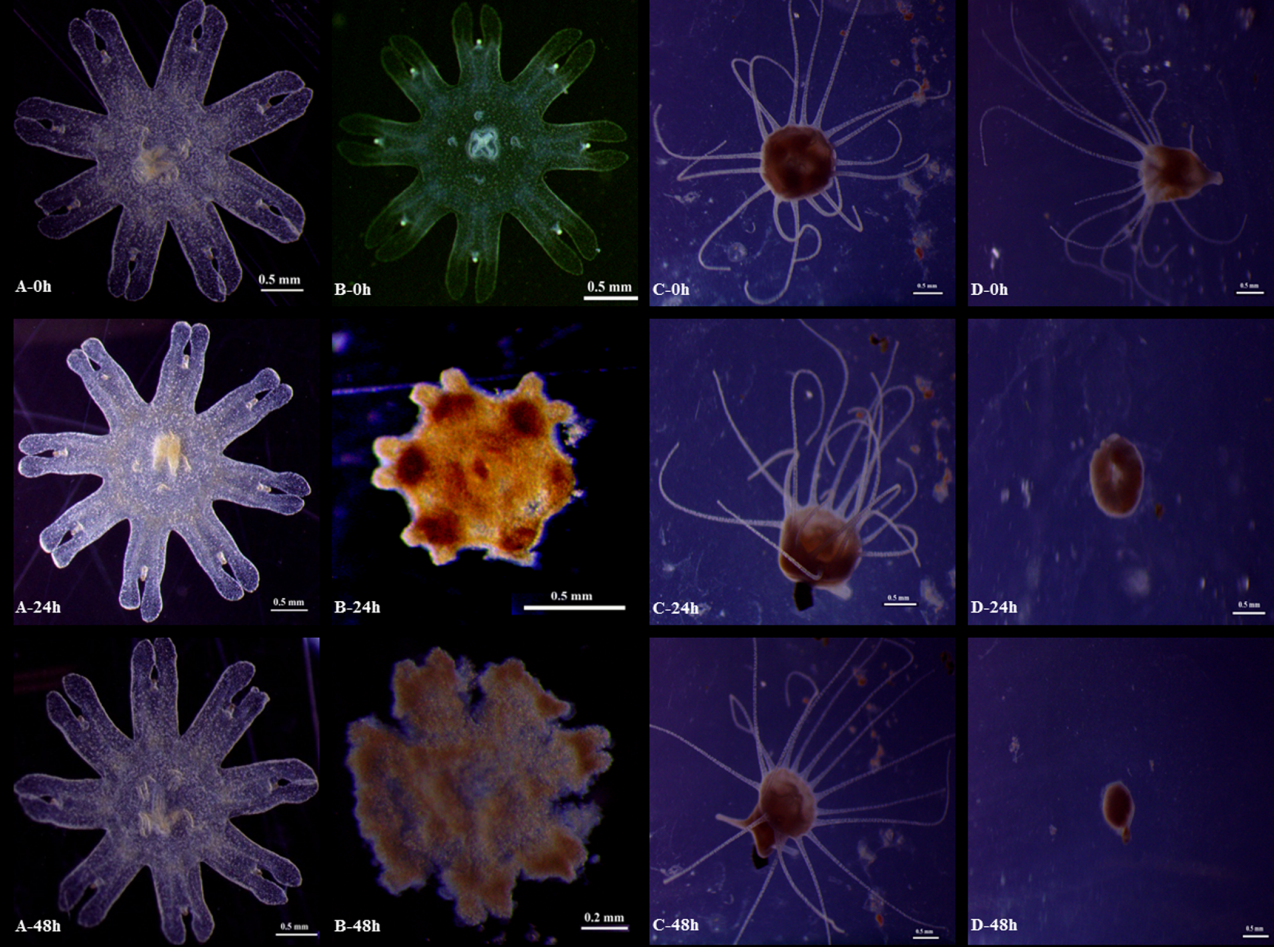
Morphological changes observed in *Aurelia* sp.1 ephyrae and polyps exposed to tea saponin. (A) Ephyrae exposed to control seawater only. (B) Ephyrae exposed to a 5 mg L^-1^ concentration of tea saponin. (C) Polyps exposed to control seawater only. (D) Polyps exposed to a 5 mg L^-1^ concentration of tea saponin.

The test results of *Aurelia* sp.1 ephyrae and polyps exposed to different concentrations of tea saponin are reported in Figs 1, 2, 3 and 4. The morphological changes in the *Aurelia* sp.1 ephyrae and polyps exposed to different concentrations of tea saponin are shown in Fig 5.

All of the control *Aurelia* sp.1 ephyrae and polyps survived after 48 h. All *Aurelia* sp.1 ephyrae showed 100% mortality at tea saponin concentrations above 5 mg l^-1^ after 24 h, and all *Aurelia* sp.1 ephyrae exposed to concentrations above 3 mg l^-1^ died after 48 h (Fig 1). However, no ephyrae died when exposed to 0.1, 0.5, and 1 mg l^-1^ tea saponin for 24 h. All *Aurelia* sp.1 polyps showed 100% mortality at tea saponin concentrations above 2 mg l^-1^ after 24 h and 48 h (Fig 2). No polyps died after 24 h and 48 h of exposure to 0.1 mg l^-1^ tea saponin.

The median lethal concentration (LC_50_) values of tea saponin and their 95% confidence limits for different exposure time are presented in Table 1. The LC_50_ values for *Aurelia* sp.1 ephyrae decreased from 1.9 mg L^-1^ after 24 h to 1.1 mg L^-1^ after 48 h of exposure based on immobility and from 0.8 mg L^-1^ after 24 h and 1.0 mg L^-1^ after 48 h of exposure based on the pulsations frequency. The LC_50_ values for *Aurelia* sp.1 polyps were 0.4 mg L^-1^ after both 24 h and 48 h of exposure based on mortality.

**Table 1:** LC_50_ (EC_50_) values of *Aurelia* sp.1 ephyrae and polyps exposed to tea saponin.

Stage	End-point	Exposure time	LC_50_ /EC_50_ (mg l^-1^)	95% confidence limits
Ephyrae	Immobility	24 h	1.9	1.7–2.1
		48 h	1.1	0.9–1.2
	Altered Pulsation Frequency	24 h	0.8	0.2–2.0
		48 h	1.0	0.6–1.3
Polyps	Mortality	24 h	0.4	0.4–0.5
		48 h	0.4	0.4–0.5

The *Aurelia* sp.1 ephyrae LOEC values calculated from the pulsations frequency were observed at the concentrations of 2 mg l^-1^ after 24 h and 2 mg l^-1^ after 48 h and of 3 mg l^-1^ after 24 h and 2 mg l^-1^ after 48 h, respectively (Fig 3). The LOEC values of the polyps calculated from mortality were observed 1 mg l^-1^ concentration after both 24 h and 48 h (Fig 4).

All of the control *Aurelia* sp.1 ephyrae had normal bread knife-like rhopalial lappets with spade-like rhopalial canals (Fig 5A). However, *Aurelia* sp.1 ephyrae exhibited signs of tissue shriveling and degradation after 24 h and 48 h of exposure to 5 mg L^-1^ tea saponin (Fig 5B). Control *Aurelia* sp.1 polyps had fully extended tentacles (Fig 5C), while polyps exposed to 5 mg L^-1^ tea saponin lost tentacles and died after 24 h and 48 h of exposure (Fig 5D).

## Discussion

In our present study, two end-points were used to calculate the LC_50_ values. The LC_50_ values of *Aurelia* sp.1 ephyrae calculated from the pulsations’ frequency were lower than those from immobility, which is consistent with the results of previous studies [20–21]. For example, the LC_50_ values for exposure to cadmium nitrate, calculated from the frequency of pulsations and immobility, were 0.1 mg L^-1^ and 0.5 mg L^-1^ respectively [20]. Furthermore, the results of exposing *Aurelia* sp.1 ephyrae to eserine and chlorpyrifos demonstrate that the frequency of pulsations is a more sensitive indicator than immobility in toxicological studies [21].

Saponin, a water-soluble glucoside, can induce swelling in the gill lamella and interlamellar epithelia, lyse blood cells, and lower the surface tension between the water and the gills in fish, thus leading to a slow death through the prevention of oxygen uptake [14–15, 25–26]. Therefore, tea seed cakes or tea saponin have been widely used in aquaculture as a piscicide to kill predators [14]. The toxic effects of tea saponin on different cultured organisms have been determined to provide qualitative data for aquaculture farmers [16–19]. Tang (1961) reported that the lethal dosages of tea saponin were 1.0 to 1.5 mg L^-1^ and could successfully eliminate undesirable fishes without injury to shrimps [16]. Terazaki et al. (1980) reported that the effective dosage of crude saponin for the eradication of predatory fishes was 1.1 mg L^-1^, a concentration at which all of the studied shrimp and crabs survived [17]. Zhu et al. (1991) reported that the lethal dosage of tea saponin for harmful fish was 0.5 to 0.7 mg L^-1^, and Shao and Chang (2004) reported that the effective dosage of tea seed cakes was 15–20 mg L^-1^ for the elimination of the hydrozoan jellyfish, *Proboscidactyla ornata* [18–19].

In the case of the moon jellyfish, *Aurelia* sp.1, similar mortality mechanisms to those of predatory fish may be at work, in that death follows damage to the tissues or oxygen deprivation. The ephyrae and polyps of *Aurelia* sp.1 showed high mortality at rather low tea saponin concentrations-the LC_50_ values were approximately 1.1 mg L^-1^ and 0.4 mg L^-1^ after 48 h exposure at a temperature of 16°C and a salinity of 31.5 ppt. Therefore, our results showed that tea saponin is a potentially effective pesticide for the control *Aurelia* sp.1 ephyrae and polyps in sea cucumber culture ponds.

However, the sensitivity of the sea cucumber *A*. *japonicus* to sea saponin should be considered when farmers use tea saponin to control *Aurelia* sp.1 ephyrae and polyps in sea cucumber culture ponds. Only one study determined the effects of tea saponin on the sea cucumber *A*. *japonicus* [27]. Zhang et al. reported that the LC_50_ value of the sea cucumber *A*. *japonicus* exposed to tea seed cake was 135.25 mg L^-1^ after 48 h, which corresponds to the tea saponin in the range of 13.5–20.3 mg L^-1^ [27]. The resistance of *A*. *japonicus* to tea saponin is 12–18 times greater than that of *Aurelia* sp.1 ephyrae. In addition to this, the safe tea saponin dosage for the sea cucumber was 1.35–2.03 mg L^-1^ based on an empirical application factor of 0.1 [28]. Therefore, the appropriate tea saponin dosage should be paid enough attention to minimize any possible damage to sea cucumbers. We recommend that the level of tea saponin used to eradicate *Aurelia* sp.1 ephyrae and polyps in sea cucumber culture ponds be lower than 1.35 mg L^-1^.

Furthermore, the physiological conditions of the sea cucumber *A*. *japonicus* at different developmental stages should be considered in future research. Chen et al. (1996) investigated the effects of saponin on the survival, growth, molting and feeding of juvenile *Penaeus japonicus* and the results showed that the maximum acceptable toxicant concentration was 0.1 mg L^-1^ saponin, which is lower than the recommended level [17, 29]. Therefore, it is necessary to investigate the differing effects of tea saponin on the survival, feeding and growth of *Apostichopus japonicus* at different developmental stages. Not only this, but more information on the effects of tea saponin on other representative organisms in the sea cucumber culture ponds is needed to verify its potential environmental impact.

In conclusion, our study first determined the toxic effects of tea saponin on the ephyrae and polyps of the moon jellyfish *Aurelia* sp.1, and significant morphological changes, behavioral abnormality and mortality were found in 24h and 48h exposure experiments. The LC_50_ values for *Aurelia* sp.1 ephyrae and polyps exposed to tea saponin were 1.1 mg L^-1^ and 0.4 mg L^-1^, respectively, based on immobility after 48 h of exposure. As such, it can be concluded that tea saponin is an effective pesticide to control *Aurelia* sp.1 in sea cucumber culture ponds. However, appropriate tea saponin dosages should be determined after taking into consideration any possible damage to the sea cucumber, due to the relatively high sensitivity of *A*. *japonicus* to sea saponin. Future research is needed to address the effects of tea saponin on *A*. *japonicus* and other organisms in sea cucumber culture ponds, in order to evaluate its potential environmental impact.

## Supporting information

S1 File The raw data for each figure. The test results of *Aurelia* sp.1 ephyrae and polyps exposed to different concentrations of tea saponin are shown in each sheet.
(XLSX)
